# A combination vaccine against SARS-CoV-2 and H1N1 influenza based on receptor binding domain trimerized by six-helix bundle fusion core

**DOI:** 10.1016/j.ebiom.2022.104297

**Published:** 2022-10-04

**Authors:** Rui Shi, Jiawei Zeng, Ling Xu, Fengze Wang, Xiaomin Duan, Yue Wang, Zheng Wu, Dandan Yu, Qingrui Huang, Yong-Gang Yao, Jinghua Yan

**Affiliations:** a CAS Key Laboratory of Pathogenic Microbiology and Immunology, Institute of Microbiology, Chinese Academy of Sciences, Beijing 100101, China; b University of Chinese Academy of Sciences, Beijing 100049, China; c Key Laboratory of Animal Models and Human Disease Mechanisms of the Chinese Academy of Sciences & Yunnan Province, and KIZ-CUHK Joint Laboratory of Bioresources and Molecular Research in Common Diseases, Kunming Institute of Zoology, Chinese Academy of Sciences, Kunming, Yunnan 650201, China; d Kunming National High-level Biosafety Research Center for Non-Human Primates, Kunming Institute of Zoology, Chinese Academy of Sciences, Kunming, Yunnan 650107, China; e Institute of Physical Science and Information, Anhui University, Hefei, 230039, China; f National Resource Center for Non-Human Primates, National Research Facility for Phenotypic & Genetic Analysis of Model Animals (Primate Facility), Kunming Institute of Zoology, Chinese Academy of Sciences, Kunming, Yunnan 650107, China; g Kunming College of Life Science, University of Chinese Academy of Sciences, Kunming, Yunnan 650204, China

**Keywords:** SARS-CoV-2, Influenza A viruses, Co-infection, Combined subunit vaccines

## Abstract

**Background:**

Increasing severe morbidity and mortality by simultaneous or sequential infections with SARS-CoV-2 and influenza A viruses (IAV), especially in the elderly and obese patients, highlight the urgency of developing a combination vaccine against COVID-19 and influenza.

**Methods:**

Self-assembling SARS-CoV-2 RBD-trimer and Influenza H1N1 HA1-trimer antigens were constructed, upon the stable fusion core in post-fusion conformation. Immunogenicity of SARS-CoV-2 RBD-trimer vaccine and H1N1 HA1-trimer antigens candidates were evaluated in mice. Protection efficacy of a combination vaccine candidate against SARS-CoV-2 and IAV challenge was identified using the K18-hACE2 mouse model.

**Findings:**

Both the resultant RBD-trimer for SARS-CoV-2 and HA1-trimer for H1N1 influenza fully exposed receptor-binding motifs (RBM) or receptor-binding site (RBS). Two-dose RBD-trimer induced significantly higher binding and neutralizing antibody titers, and also a strong Th1/Th2 balanced cellular immune response in mice. Similarly, the HA1-trimer vaccine was confirmed to exhibit potent immunogenicity in mice. A combination vaccine candidate, composed of RBD-trimer and HA1-trimer, afforded high protection efficacy in mouse models against stringent lethal SARS-CoV-2 and homogenous H1N1 influenza co-infection, characterized by 100% survival rate.

**Interpretation:**

Our results represent a proof of concept for a combined vaccine candidate based on trimerized receptor binding domain against co-epidemics of COVID-19 and influenza.

**Funding:**

This project was funded by the Strategic Priority Research Program of CAS (XDB29040201), the National Natural Science Foundation of China (81830050, 81901680, and 32070569) and China Postdoctoral Science Foundation (2021M703450).

**Research in context**

**Evidence before this study**

Due to disparities in global access to vaccines, waning immunity after vaccination or infection, virus variants that evade immune protection, and re-entries from zoonotic reservoirs, an expectation that SARS-CoV-2 will disappear is still unrealistic. Retrospective studies reported that co-infecting SARS-CoV-2 and influenza A virus (IAV), another major pathogen that principally infects the human respiratory system, enhances the severity and mortality of pneumonia in the elderly, comorbid, and obese patients. A potency combination vaccine is a promising countermeasure against SARS-CoV-2 and IAV co-infection.

**Added value of this study**

Recently published studies proved that RBD-based vaccines are applicable to combat SARS-CoV-2. Meanwhile, the World Health Organization (WHO) recommends the seasonal influenza vaccine for routine infants and elders to reduce the risk of IAV infection. Here, we designed non-tagged SARS-CoV-2 RBD-trimer and influenza H1N1 HA1-trimer antigens. A combination of those two immunogens adjuvanted with saponin-based adjuvant protects against SARS-CoV-2 infection in the lungs and stringent lethal challenges of pandemic influenza virus co-infection in mouse models.

**Implications of all the available evidence**

These promising data support the combination vaccine candidate for further clinical development to control future co-epidemics of COVID-19 and H1N1 influenza.

Alt-text: Unlabelled box

## Introduction

The ongoing COVID-19 pandemic, caused by SARS-CoV-2, has placed great tension globally and affected almost all aspects of human endeavors.^1^ Although the ongoing global effort in the deployment of effective vaccines in many countries may bring the COVID-19 pandemic under control, an expectation that SARS-CoV-2 will quickly disappear is still unrealistic.^1^ A large number of SARS-CoV-2 breakthrough infection cases in fully vaccinated individuals warrant a possible regular boost shot of COVID-19 vaccines every year.^1^^,^^2^ Nowadays, influenza A virus (IAV) and Coronavirus (CoVs) are major pathogens that principally infect human respiratory system.^3^ Seasonal influenza epidemics caused 3 million to 5 million severe cases and 300,000 to 500,000 deaths globally each year, according to the World Health Organization (WHO). A retrospective study based on serological analysis reported that the coinfection rate of SARS-CoV-2 and IAV during the COVID-19 outbreak at the end of 2019 was as high as 49.8%.^4^ Case study analysis also certified that influenza increased SARS-CoV-2 transmission by 2 to 2.5 folds,^5^ and their co-infections led to a 5.9-time greater risk of death, especially in the elderly and obese patients.^6^ To date, no clinical effective prophylactics are available for the prevention of both SARS-CoV-2 and IAV infections, highlighting an urgent need for combination vaccine development.

Currently, several clinically approved COVID-19 vaccines, including traditional inactivated vaccines (CoronaVac^7^ and BBIBP-CorV^8^), adenoviral vectored vaccines (AZD1222, Ad26.COV2.S, and Gam-COVID-Vac),^9^ and mRNA vaccines (mRNA-1273 and BNT162b2),^10^ have relieved some public health burden by greatly reducing disease morbidity and mortality rate.^1^^,^^2^ However, inactivated vaccines suffered weak immunogenicity, especially in the elderly.^2^ Some severe side effects including thrombosis with thrombocytopenia syndrome and myocarditis have been shown to be related to adenoviral vectored vaccines and mRNA vaccines, respectively.^2^ By contrast, with less safety concern and potent immunogenicity, adjuvanted protein subunit vaccine might be a superior approach for long-term application as a routine vaccination regimen. For influenza, the most widely used vaccine is the egg-based split-virus vaccine.^11^ However, vaccine effectiveness can be compromised by antigenic changes from virus egg-adaptive mutations.^12^ Moreover, a shortage of enough egg resources to cover the global population is another major worrisome.^12^ Obviously, protein subunit vaccine also provides a better choice.

The membrane-anchored fusion trimer glycoproteins from SARS-CoV-2 and influenza viruses, belonging to the families of Orthomyxoviridae and Coronaviridae, respectively, have been classified as class I fusion protein based on their similarities in structure and function.^13^ In general, class I fusion protein contains two functional subunits.^13^^,^^14^ One is a receptor-binding subunit that is responsible for engaging human cellular receptors and initiating virus cellular infection. It has been shown that SARS-CoV-2 receptor-binding domain (RBD), located at the C-terminus of the spike S1 subunit, is immunodominant, comprises multiple distinct antigenic sites, and is the target of approximately 90% of the neutralizing activity present in COVID-19 convalescent sera.^15^, ^16^, ^17^, ^18^ Correspondingly, for the influenza virus, as a receptor-binding subunit of HA, HA1 is also the immunodominant domain of the protein and is the target of most neutralizing antibody responses induced by IAV vaccines or infection.^11^ Those observations motivate the use of SARS-CoV-2 RBD and influenza virus HA1 as the vaccine immunogen. However, monomeric RBD and HA1 proteins stand a shortcoming of low immunogenicity, mostly arising from their relatively small molecular sizes.^19^ Various strategies of multimerization have been shown to markedly enhance humoral immune responses to target pathogens. These include fusing RBD with human IgG Fc^20^ or T4 trimerization tag,^21^ and heterologous self-assembling protein platforms for an extensive display of targeting antigens such as the SpyCatcher-SpyTag technology,^22^ a two-component nanoparticle system with I53-50A and I53-50B,^23^ or chemical conjugation to virus-like particles.^24^ However, these approaches inevitably induce exogenous proteins into immunogens which may raise some additional vaccine safety concerns.^19^ Moreover, the pre-existing exogenous protein-targeted antibodies also potentially compromised or interfered with the immune responses following inoculations with other vaccines developed by the same vaccine platform.^25^, ^26^, ^27^ In contrast, a strategy of multimerization without inducing an exogenous protein or tag appears to be a superior choice. The other functional subunit of class I fusion protein is a fusion-mediating subunit (S2 subunit for SARS-CoV-2 S and HA2 subunit for influenza HA protein) that drives the fusion of virus and cell membranes.^13^^,^^14^ It has been well established that upon engagement with a target cell, class I fusion protein irreversibly transits from a metastable prefusion form to its lowest-energy stable post-fusion conformation through a dramatic conformational rearrangement.^13^^,^^14^ During that process, a critical stable six-helix bundle fusion core is formed. In detail, the six-helix bundle fusion core is constituted by heptad repeats (HRs), HR1 and HR2 from SARS-CoV-2 S2 subunit, or by the long alpha helix (LAH) from the influenza HA2 subunit.^28^^,^^29^ Moreover, both HRs and LAH exhibit a strong hydrophobic face that facilitates the formation of their stable homotrimers in the fusion core.

In this study, taking advantage of self-assembling stable homotrimers of SARS-CoV-2 fusion core in post-fusion form, we firstly constructed SARS-CoV-2 RBD-trimer without introducing an exogenous protein or tag. We found that RBD-trimer elicited significantly higher neutralizing antibody titers than RBD-monomer, RBD-dimer, and spike ectodomain trimer (S-ECD-trimer), and also a Th1/Th2 balanced cellular immune response in mice. Theoretically, our non-tagged trimerization strategy is also effective when applied to other class I fusion viral vaccines including IAV. As expected, a resultant HA1-trimer vaccine candidate based on H1N1 A/California/07/2009 influenza also induced robust neutralizing antibodies. We further developed a combination vaccine candidate by mixing RBD-trimer and HA1-trimer and found that it afforded protection against both SARS-CoV-2 and lethal homogenous H1N1 influenza challenge, suggesting promise for its further clinical assessment.

## Methods

### Cells and viruses

HEK293T cells (ATCC, CRL-3216), Vero E6 cells (ATCC, CRL-1586), and Madin-Darby canine kidney (MDCK) cells (ATCC, CCL-34) were cultured at 37°C in Dulbecco's Modified Eagle medium (DMEM) with 10% fetal bovine serum (FBS). SARS-CoV-2 (hCoV-19/Beijing/CAS-B001R/2020, Accession ID: EPI_ISL_514257) was isolated by Institute of Microbiology, Chinese Academy of Sciences. Vero E6 cells were applied to the reproduction of SARS-CoV-2 stocks. H1N1 A/California/07/2009 was propagated in specific pathogen-free (SPF) embryonated eggs (Beijing Merial Vital Laboratory Animal Technology). For SARS-CoV-2 and IAV co-infection experiments, we also used a SARS-CoV-2 virus strain provided by Guangdong Provincial Center for Disease Control and Prevention, Guangdong Province of China, which was described in our previous studies.^30^^,^^31^ Cell lines were authenticated by short tandem repeat profiling.

### Gene construction

The genes encoding SARS-CoV-2-RBD-monomer (S protein residues 319-537, GenBank accession number: EPI_ISL_402119), SARS-CoV-2-RBD-dimer (two S protein residues 319-537 connected as tandem repeat^19^), SARS-CoV-2-RBD-trimer (S protein residues 918-966, a 22-amino-acid linker LVPRGSGGSGGSGGLEVLFQGP, 1163-1203, and 319-537), SARS-CoV-2 S-ECD-trimer (S protein residues 13-1213 with two proline substitutions at residues 986 and 987, a “GSAS” substitution at residues 682-685, a trimerization folding motif and a protease cleavage site^32^), HA1-trimer (HA protein residues 403-474 and 18-340, GenBank accession number: FJ966974.1), HA-ECD-trimer (HA protein residues 18-529 with a thrombin cleavage site and a trimerization folding sequence^33^), RBD-IAV-fusion core-trimer (S protein residues 319-537 and HA protein residues 403-474), HA1-SARS-CoV-2-fusion core-trimer (HA protein residues 18-340 and S protein residues 918-966, a 22-amino-acid linker LVPRGSGGSGGSGGLEVLFQGP, S protein residues 1163-1203), and hACE2-ECD (residues 18-615, GenBank accession number: BAJ21180), were cloned into the pCAGGS expression vector (Addgene) using the *EcoR*I and *Xho*I restriction sites with N-terminal self-signal peptides and C-terminal 6 × His tag. The variable regions of 5J8 (PDB accession number: 4M5Y)^34^ and 32D6 (PDB accession number: 6A4K)^35^ were linked with the coding sequences for the human IgG_1_ constant region to generate full-length mAbs.

### Protein expression and purification

The recombinant proteins contained in the pCAGGS vector were expressed in HEK293T cells. After transfection for about three days, the supernatants of the cells were collected. The antigen and mAb proteins were initially isolated by the Histrap Hp or Hitrap Protein A 5 mL column (GE Healthcare). Then, the samples were further purified by molecular size using the Superdex 200 column (GE Healthcare) in a buffer composed of 20 mM Tris-HCl (pH 8.0) and 150 mM NaCl. The purity of the protein was determined by SDS-polyacrylamide gel electrophoresis (SDS-PAGE).

All of the antibodies have been validated in previous studies both by binding to SARS-CoV-2 spike or influenza HA1, and when applicable, have been confirmed to give similar results as that described in publications by other groups.

### Analysis of protein expression

A double-antibody sandwich enzyme-linked immunosorbent (DAS-ELISA) assay was employed to determine the expression levels of antigens.^36^ 200 ng of the CB6-Fab protein (an RBD-specific capture antibody) was coated in a microtiter plate overnight. After blocking for one hour at room temperature, 100 μL gradient concentrations of purified RBD-monomer, RBD-trimer, S-ECD-trimer proteins (from 2 μg/mL to 2 ng/mL), or 8-fold diluted supernatant to be measured were added to the plates and incubated for two hours. After washing three times, 200 ng mAb GH12 protein (an RBD-specific antibody that does not compete with CB6, PDB accession number: 7D6I) was used as a secondary antibody to contact the proteins. Following incubation and washing, an HRP-labeled anti-human Fc antibody (Sino Biological) was added. The plates on a microplate reader are set to 450 nm for HRP-based substrate development. The standard curve was fitted in a sigmoidal, 4-PL, and X is the concentration model by the exponential form of standard protein concentration and absorbance value. Then, the titers of antigens were obtained by 4-PL curves [Equation: y = A2+(A1-A2)/(1+(X/X0)^P)].^36^ The characteristics of 4-PL curves are listed: A1, A2, X0, and P indicated the estimation of asymptotes under curves, the estimation of asymptotes on curves, and the concentration for 50% of maximal effect, and the slope of the curve. For HA-related antigens, HA1-specific mAb 5J8-Fab and full-length 32D6 were used for assays.

### Analytical ultracentrifugation

SARS-CoV-2-RBD-trimer and HA1-trimer were analyzed in the buffer of 20 mM Tris (pH 8.0) and 150 mM NaCl. Sedimentation velocity experiments were performed in a ProteomeLab XL-I analytical ultracentrifuge (Beckman Coulter), equipped with the AN-60Ti rotor and conventional double-sector aluminum centerpieces of 12 mm optical path length. Before the run, the rotor was equilibrated for approximately one hour at 20°C. Then, experiments were carried out at 20°C and 31,000 rpm, using continuous scan mode and radial spacing of 0.003 cm. Scans were collected in intervals of three minutes at 280 nm. The fitting of absorbance versus cell radius data used SEDFIT software (sedfitsedphat.nibib.nih.gov) and the continuous sedimentation coefficient distribution c(s) model, covering a range of 0-15 S.

### Surface plasmon resonance (SPR)

SPR assays were performed with BIAcore 8K (GE Healthcare) at 25°C. The running buffer was 10 mM Na_2_HPO_4_, 2 mM KH_2_PO_4_ (pH 7.4), 137 mM NaCl, 2.7 mM KCl, and 0.005% Tween 20. SARS-CoV-2-RBD-monomer or trimer protein was immobilized on the CM5 biosensor chip (GE Healthcare) at about 3000-5000 response units. Gradient concentrations of hACE2 protein (from 12.5 to 200 nM for SARS-CoV-2-RBD-monomer and from 6.25 to 100 nM for SARS-CoV-2-RBD-trimer) flowed through the chip surface at a rate of 30 μL/min. After each cycle, 10 mM NaOH was used to regenerate the sensor surface.

To measure the affinity of HA1-trimer and HA-specific mAbs, 5J8 or 32D6 protein was captured via a Protein A biosensor chip (GE Healthcare). HA1-trimer protein (from 0.78 to 12.5 nM) flowed through the chip surface at a rate of 30 μL/min. After each cycle, Gly-HCl (pH 1.7) was employed to regenerate the sensor surface. The binding kinetics was fitted in a 1:1 binding model using BIAevaluation software (GE Healthcare).

### Differential scanning fluorimetry (DSF)

Prometheus NT.48 instrument (NanoTemper Technologies) was used to determine melting temperatures for adjuvanted HA1-trimer and RBD-trimer protein. Unlabeled 10 µL samples (at a concentration of 1 mg/mL) were filled in a glass capillary (NanoTemper Technologies) and placed on to the sample holder. A temperature gradient of 1°C/min from 25°C to 95°C was applied to determine the intrinsic protein fluorescence at 330 nm, 350 nm, and a ratio of 350/330 nm. Each group of samples was repeated three times.

### Ethics statement

This study was approved and conducted in strict accordance with the recommendations stated in the Guidelines for the Care and Use of Laboratory Animals issued by the Ethics Committee of the Institute of Microbiology and Kunming institute of zoology, Chinese Academy of Sciences.

### Vaccine formulation

MA103 adjuvant is a liposome of 4 mg/mL DOPC (Sigma), 2 mg/mL Pod Sterol (Sigma), and 0.2 mg/mL QS-21 (MaxHealth Biotech LLC) through membrane extrusion and aseptic filtration. The medium particle size is 90-110 nm, and the endotoxin content is less than 2.5 Eu/mL. For the immunization of mice, antigen proteins (1 mg/mL) and an equal volume of MA103, aluminum hydroxide (Thermo Scientific), aluminum hydroxide MPs (Institute of Process Engineering, Chinese Academy of Sciences), or MF59-like adjuvant (MaxHealth Biotech LLC) were mixed uniformly by vortex oscillation.

### Enzyme-linked immunosorbent assay (ELISA)

200 ng per well of SARS-CoV-2-RBD-monomer protein in 50 mM carbonate-bicarbonate buffer (pH 9.6) were coated for RBD-specific antibody titers detection. Similarly, HA1-monomer was used to detect HA1-specific antibody titers. After blocking with 5% skim milk at 37°C for one hour, the plates were incubated with 100 μL two-fold serially diluted mice serum at 37°C for another one hour. The HRP-labeled anti-mouse Fc secondary antibody (Yeasen) was added after washing the plates three times. Then, 50 μL 3, 3’, 5, 5’-Tetramethylbenzidine (Beyotime Biotechnology) was used as the substrate and the reactions were stopped with 50 μL 2 M sulphuric acid. A microplate reader (PerkinElmer) was employed to measure the absorbance at 450 nm. The endpoint antibody titers were defined as the highest reciprocal dilution of serum to yield 2.1 times higher than the optical absorbance value (OD_450_) of the background values.

For detecting the RBD-specific, HA1-specific, and fusion core-specific antibody titers, 200 ng per well of fusion core protein (HA1-SARS-CoV-2-fusion core-trimer or RBD-IAV-fusion core-trimer), RBD-monomer, RBD-trimer, HA1-trimer, or His-tag (hACE2-ECD) protein were employed.

### Intracellular cytokine staining (ICS) and flow cytometry

An ICS assay was performed to characterize antigen-specific CD4+ T cell immune responses.^16^ Briefly, mouse splenocytes of immunized mice were transferred to the 96-well plates (1 × 10^6^ cells/well) and stimulated with the peptide pool (2 μg/mL of individual peptide) (Bai Tai) for four hours. The cells were incubated with Golgiplug (BD Biosciences) for an additional 12 hours at 37°C. Then, the cells were harvested and stained with PE-anti-CD3, and FITC-anti-CD4 surface markers (BD Biosciences). Subsequently fixed and permeabilized in permeabilizing buffer (BD Biosciences), the splenocytes were incubated with BV421-anti-IFN-γ, BV605-anti-IL-2, BV786-anti-IL-4, or PerCP Cy5.5-anti-TNF-α antibodies (BD Biosciences). Cell sorting and flow cytometric analysis were performed via FACSAria III flow cytometer (BD Biosciences) and the data were analyzed using FlowJo 7.6.1.

### SARS-CoV-2 neutralization assay

The neutralization assay was conducted in a BSL-3 facility. Briefly, two-fold diluted inactivated serum was incubated with 100 TCID_50_ SARS-CoV-2 at 37°C for one hour. The mixture was transferred to the monolayer of pre-inoculated Vero E6 cells in 96-well plates. Then, the plates were incubated at 37°C for three days, following which the CPE of the virus was observed microscopically at 40-fold magnification. The neutralization titers were defined as the reciprocal of serum dilution required for 50% neutralization of viral infection.

### H1N1 A/California/07/2009 neutralization assay

The neutralization assay was conducted in a BSL-2 facility. MDCK cells were seeded in 96-well plates, at 15,000 cells per well in DMEM medium supplemented with 1% FBS. Duplicate serial dilutions of heat-inactivated (30 min at 56°C) serum samples were prepared in DMEM medium without trypsin/EDTA and mixed with H1N1 A/California/07/2009 virus in assay medium containing trypsin/EDTA, for one hour at 37°C, 5% CO_2_. The mixture was subsequently transferred to the MDCK cells at a final concentration of 100 TCID_50_ viruses per well and incubated for 72 hours at 37°C, 5% CO_2_. The positive control was the mixture of viruses and cells, while the negative control was only cells. Cells were fixed with 80% acetone for 10 minutes and air-dried. After washing with buffer solution, the cells were incubated with rabbit anti-influenza A nucleoprotein (Abcam) for one hour. According to the conventional ELISA, the neutralization titers were defined as the reciprocal of the maximum dilution multiple, which according to optical absorption value at 450 nm is less than half the difference between positive and negative control value, then plus negative control value.

### Hemagglutination inhibition (HAI)

HAI of immunized mice were monitored as previously described.^37^ Serum samples from immunized animals were treated with receptor destroying enzymes (Denka Seiken). The two-fold serially diluted serum samples were mixed with the H1N1 A/California/07/2009 virus (4 HA_100_). After incubating at 37°C for 30 min, the mixture was transferred to the V-bottom 96-well microtiter plates and added 1% turkey erythrocytes incubated at room temperature for 20 min. The HAI titers were defined as the reciprocal of the highest serum dilution required for complete hemagglutination inhibition.

### Mice immunizing and viruses challenging

Ideally, a priori power analysis should be performed to determine the appropriate sample size in each group, sample size = 2(Z^α^_/2_+Z^β^)^2^ × P(1−P)/(p_1_−p_2_)^2^, Z^α^_/2_ = Z_0.05/2_ = Z_0.025_ = 1.96 at type 1 error of 5%, Z^β^ = Z_0.20_ = 0.842 at 80% power, p_1_−p_2_ = Difference in proportion of events in two groups, P = Pooled prevalence = (prevalence in case group [p_1_]+prevalence in the control group [p_2_])/2. p1 = 0, p2 = 100.^42^ In this study, sample size = 2(1.96+0.842)^2^ × 0.5(1−0.5)/(-1)^2^ = 3.93. Therefore, more than 4 mice in each group are sufficient to meet the requirement of statistical power. To reduce the error and improve the reliability of the results, we generally used 6-10 mice per group following the previous studies^38^, ^39^, ^40^ and the suggestions of *in vivo* experiments.^41^ 6-10 mice per group had sufficient statistical power for making a valid test according to the sample size calculation described by Charan and Kantharia.^42^ Our experimental program has been approved by the Research Ethics Committee of the Institute of Microbiology, Chinese Academy of Sciences, and complied with all relevant ethical regulations regarding animal research. The experimental operation caused no unnecessary harm to animals. In mice immunizing and viruses challenging studies, the animals were randomly divided into each group according to body weight.

For SARS-CoV-2 challenge experiments, ten hACE2 transgenic mice (HACE2-KI/NIFDC 8-10-week-old female mice from National Institutes for Food and Drug Control) in each group were intramuscularly immunized in a prime-boost regime. Phosphate-buffered saline (PBS) was employed in the negative control group. Two weeks post the second immunization, mice were infected with 5 × 10^5^ TCID_50_ of SARS-CoV-2 via the intranasal infection. All mice were euthanized on the 5th day following the challenge. Lung tissues were harvested for virus load detection (seven mice per group) and pathological examination (three mice per group).

For IAV H1N1 A/California/07/2009 challenge experiments, a prime-boost vaccination regime was performed in BALB/c mice (*n* = 10 in each group) via the intramuscular immunizations route. PBS was employed in the negative control group. Four weeks post initial immunization, mice were intranasally infected with 1 × 10^4.8^ TCID_50_ of H1N1 A/California/07/2009. Survival and weight loss were monitored daily for 14 days.

For SARS-CoV-2 and IAV co-infection experiments that were performed in a BSL-3 facility in Kunming Institute of Zoology, groups of 8-week-old K18-hACE2 mice (GemPharmatech Co., Ltd) (*n* = 8 in each group) were immunized twice with placebo, RBD-trimer, HA1-trimer, or combined vaccines, respectively. Two weeks post the second immunization, mice were infected with 1 × 10^5^ TCID_50_ H1N1 A/California/07/2009 and 1 × 10^2^ TCID_50_ SARS-CoV-2 virus. Changes in body weight and survival were monitored daily for 14 days.

### Determination of virus titer in lung tissue samples

The lung tissues of challenged mice were homogenized in 1 mL medium and clarified by low-speed centrifugation at 4500 g for 30 min at 4°C. The automated nucleic acid extraction system (TIAN LONG) was used to extract viral RNA from a 50 μL sample according to the manufacturer's instructions. OFR1ab-F: 5’-CCCTGTGGGTTTTACACTTAA-3’, OFR1ab-R: 5’-ACGATTGTGCATCAGCTGA-3’, Probe-ORF1ab: 5’-the FAM-CCGTCTGCGGTATGTGGAAAGGTTATGG-BHQ1-3’ targeting ORF1ab gene were used to detect viral RNA. The amplification was performed as followed: 42°C for 5 minutes, 95°C for 10 seconds followed by 40 cycles consisting of 95°C for 3 seconds, 60°C for 30 seconds, and a default melting curve step in an Applied Biosystems QuantStudio 5 Real-Time PCR System. The limit of detection in this RT-PCR method is 40 RNA copies per reaction mixture.

### Histopathology analysis

Three mice in each group were euthanized on the 5th day after infection according to standard procedures. Lung samples from challenged mice were collected and immobilized in 10% neutral buffer formaldehyde and embedded in paraffin wax. Tissue sections were treated with hematoxylin and eosin (H&E) and analyzed microscopically.

### Statistical analysis

Biological replicates and presentations displayed on graphs represent the mean ± SEM. We verified the assumption of normality using Shapiro-Wilk test and used one-way ANOVA to analyze the data based on the assumption of normality. Statistical significance was analyzed using an unpaired Student's t-test for two groups, an ordinary one-way ANOVA analysis of variance with multiple comparison tests for multiple groups, and a Mantel-Cox log-rank test for survival curves. All analyses were performed using GraphPad Prism 8.0. No data exclusion was performed.

### Role of the funders

The funders of this study had no role in study design, sample collection, data collection, data analyses, interpretation, or writing of the report.

## Results

### Construction and characterization of non-tagged SARS-CoV-2 RBD-trimer

**Figure 1:**
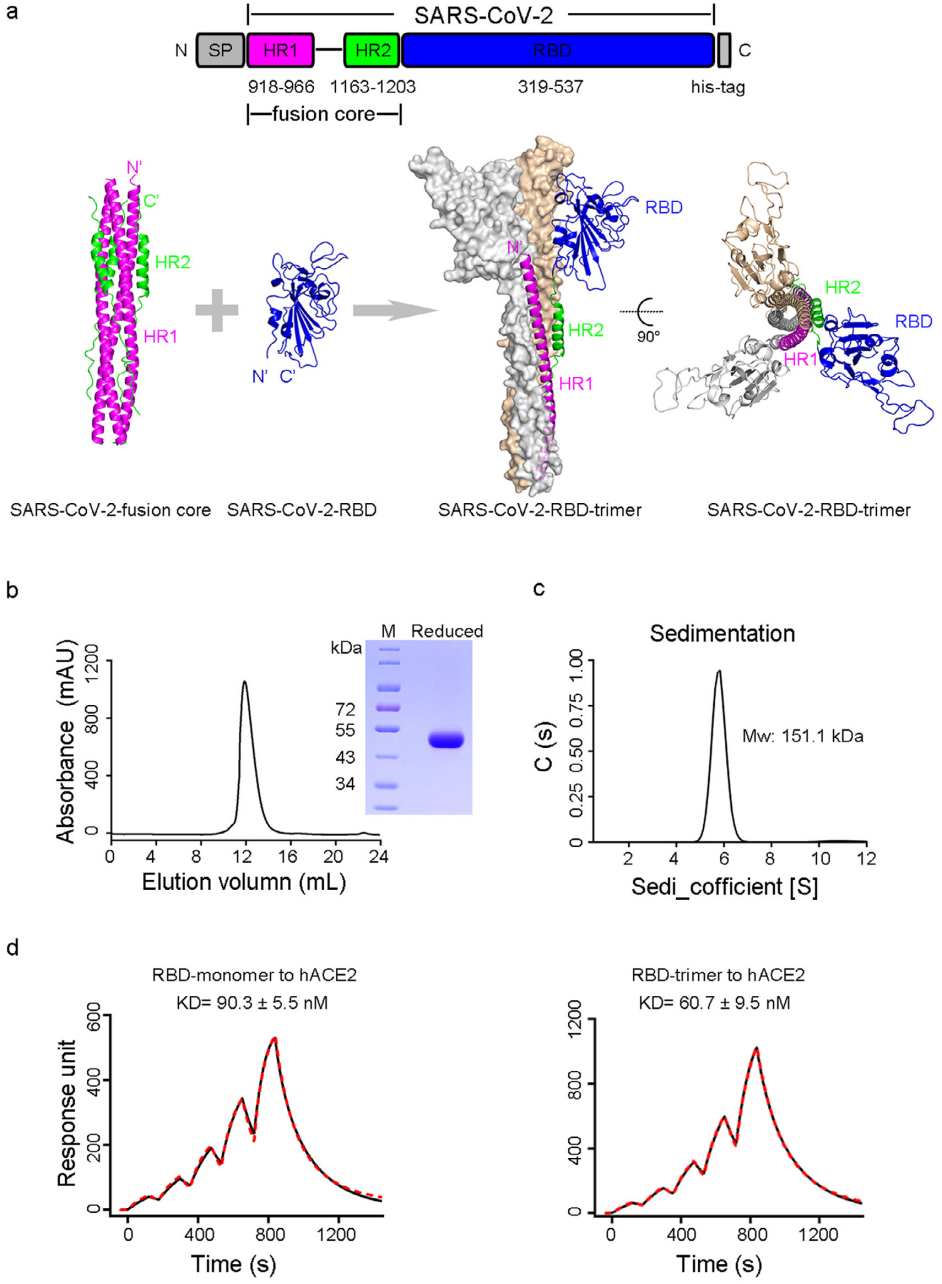
**Engineering and characterizing of SARS-CoV-2 RBD-trimer. a**, Schematic diagram of the SARS-CoV-2-fusion core and SARS-CoV-2-RBD to combine as a trimeric molecule. The upper panel shows the construction of the RBD-trimer. The lower panel shows the expected structures of the assembled trimeric molecule. **b**, Size-exclusion chromatogram and SDS-PAGE analyses of RBD-trimer. The cell supernatant was sequentially purified with Histrap excel and Superdex 200 Increase 10/300 GL column in PBS. The molecular in the single peak of gel filtration profile was shown in reducing SDS-PAGE. **c,** Ultracentrifugation sedimentation profiles of RBD-trimer. **d,** The binding kinetics profiles of monomeric or trimeric RBD to hACE2 were assessed using a single-cycle model. Antigens were captured on the chip while serial dilutions of hACE2-ECD proteins then flowed over the chip surface. The kinetic parameters were labeled accordingly. Values represent mean ± SEM of three independent assessments.

The fusion core of HR1 and HR2 with a peptide linker has been shown to efficiently self-assemble into a homotrimer with high stability.^28^ We designed SARS-CoV-2 RBD-trimer by connecting in tandem the HR1-linker-HR2 to SARS-CoV-2-RBD (Figure 1a). To avoid potential instability caused by free cysteine residue, SARS-CoV-2-RBD was truncated at K537, the position just before C538 (Figure 1a). RBD-trimer was transiently expressed in mammalian HEK293T cells. The supernatant of the transfected cells was collected for further sequential purification of affinity and gel filtration chromatography. Analytical gel filtration showed that purified RBD-trimer was eluted as a single peak with a size of ∼170 kDa (Figure 1b), revealing the trimeric form of RBD. SDS-PAGE demonstrated that RBD-trimer under reducing conditions migrated at a molecular weight (MW) of ∼50 kDa, higher than expected MW (37 kDa), implying glycosylated modifications of the protein (Figure 1b). The MW of the RBD-trimer was further corroborated as 151.1 kDa by analytical ultracentrifugation, indicating the trimeric assembly state of RBD as well (Figure 1c). A DAS-ELISA assay demonstrated that RBD-trimer reached a high expression level of 12.1 mg/L by transient transfection, higher than that of S-ECD-trimer (4.2 mg/L), suggesting a potential of high scalable production and feasibility to meet vaccine demands worldwide (Figure S1a and c). Notably, SPR assay demonstrated that the RBD-trimer bound to hACE2 with a KD of 60.7 ± 9.5 nM, which is comparable to that of the RBD-monomer (90.3 ± 5.5 nM) (Figure 1d), indicating that RBD-trimer accurately recovered the native conformation of SARS-CoV-2 receptor binding motifs (RBM).

### Immunogenicity of SARS-CoV-2 RBD-trimer vaccine candidate in animals

**Figure 2:**
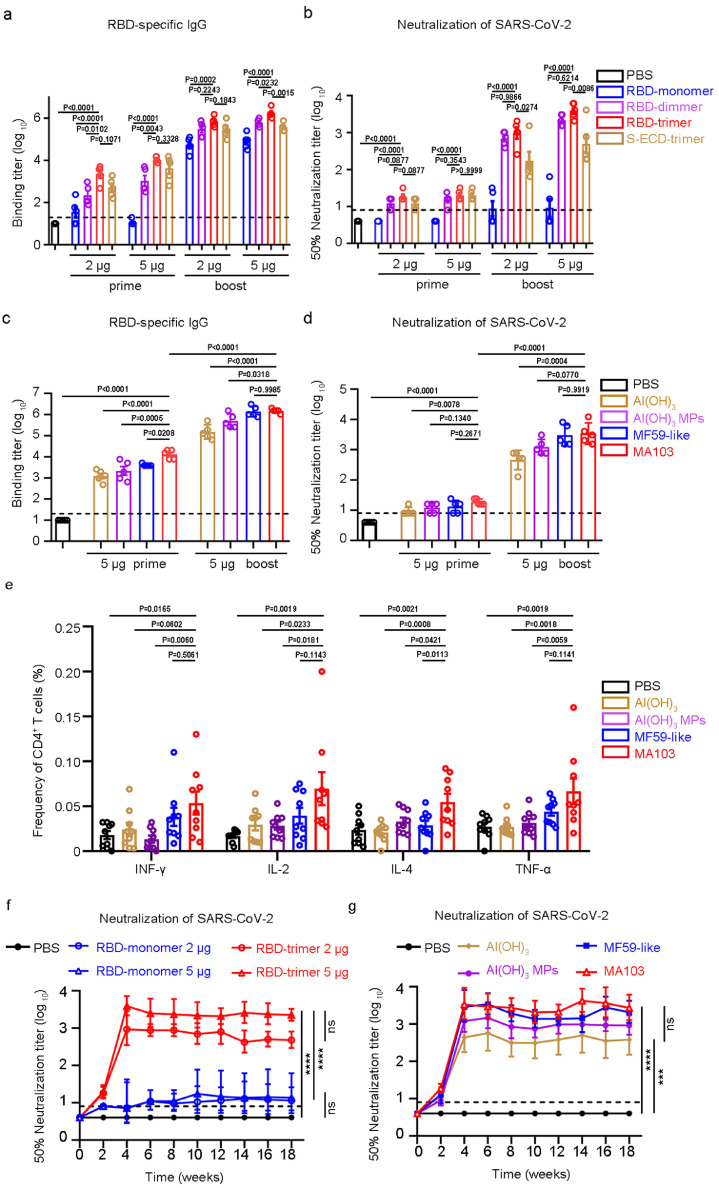
**Immunogenicity of RBD-trimer vaccine candidate in mice. a-b,** Groups of 8-week-old female BALB/c mice (*n* = 5 per group) were immunized with MA103-adjuvanted RBD-monomer, RBD-dimer, RBD-trimer, or S-ECD-trimer. PBS formulated with adjuvant was given as control. 14 days post 1st and 2nd immunizations, serum of mice was collected to evaluate the humoral response to antigens. ELISA and live SARS-CoV-2 neutralization assay show the RBD-specific IgG binding titers (a) and neutralization titers (b). **c-d,** Groups of 8-week-old female BALB/c mice (*n* = 5 per group) received 5 μg RBD-trimer protein in Al(OH)_3_, Al(OH)_3_ MPs, MF59-like, or MA103 adjuvant in a prime-boost regime. PBS formulated with adjuvant was given as control. After 14 days post priming and boosting, immune serum was collected to evaluate the humoral response to antigens. ELISA and live SARS-CoV-2 neutralization assay show the binding titers of RBD-specific IgG (c) and neutralization titers (d). **e,** Splenocytes were collected and stimulated by specific peptide pools. Intracellular cytokines (IFN-γ, IL-2, IL-4, and TNF-α) of CD4+ T cells were measured by flow cytometry assays (*n* = 9) (e). The values are representative of mean ± SEM. P values were analyzed with ordinary one-way ANOVA.

To compare the relative immunogenicity of SARS-CoV-2 multiple immunogens, eight-week-old female BALB/c mice (*n* = 5 per group) were immunized intramuscularly (IM) with 2 or 5 µg MA103-adjuvanted RBD-monomer, RBD-dimer, RBD-trimer, or S-ECD-trimer that harbors two proline substitutions at residues 986 and 987 to stabilize spike protein in the prefusion conformation. MA103 is a saponin-based adjuvant, similar to Novavax adjuvant Matrix-M™ used in the COVID-19 NVX-CoV2373 vaccine.^43^ PBS was used as a negative control. Mice were boosted with the same dose of immunogen (2 or 5 µg per mouse) two weeks later. Serum samples were collected at two weeks following either vaccination and were subjected to ELISA and authentic SARS-CoV-2 neutralization assay to determine RBD-specific IgG and SARS-CoV-2-neutralizing geometric mean titers (GMTs), respectively. As a result, the prime immunization with RBD-dimer, RBD-trimer, or S-ECD-trimer elicited comparable dose-dependent RBD-specific IgG endpoint titers and neutralizing GMTs, whereas RBD-monomer is poorly immunogenic and induced nearly undetectable RBD-specific IgG and neutralizing GMTs at both doses (Figure 2a-b). A boost immunization resulted in a substantial increase in RBD-specific IgG for all four immunogens (Figure 2a). The IgG titer elicited by RBD-trimer at the dose of 5 µg rose to 1,723,323, significantly higher than those of RBD-monomer (81,687), RBD-dimer (602,152), and S-ECD-trimer (375,059) (Figure 2a). Additionally, RBD-targeting IgG accounted for approximately 87.5% of RBD-trimer-binding antibodies, implying an immunodominance of RBD in RBD-trimer immunogen (Figure S2a). Consistent with the RBD-specific IgG titers, the neutralizing GMTs in mice receiving two high-dose injections of RBD-trimer approached 3,959, whereas neutralizing GMTs elicited by RBD-monomer, RBD-dimer, and S-ECD-trimer were 10, 2,147, and 500, respectively (Figure 2b), indicating superior immunogenicity of RBD-trimer compared to RBD-monomer, RBD-dimer, and S-ECD-trimer. Given that adjuvants play a critical role in enhancing immunity to protein subunit vaccines, we further assessed the immunogenicity of RBD-trimer in various adjuvant formulations. BALB/c mice (*n* = 5 per group) were immunized in a prime-boost regimen with 5 µg RBD-trimer in combination with one of the four adjuvants: aluminum hydroxide (Al(OH)_3_), aluminum hydroxide microparticles (Al(OH)_3_ MPs),^44^ MF59-like, or MA103. RBD-specific IgG titers were detected in all adjuvant groups post the prime vaccination, and MA103 elicited the highest magnitude (Figure 2c). Of note, MA103 induced a detectable neutralizing response in all five mice, whereas there were 4/5, 2/5, and 2/5 mice from Al(OH)_3_, Al(OH)_3_ MPs, and MF59-like groups, respectively, were scored as negative for neutralizing activity in authentic SARS-CoV-2 neutralization assay (Figure 2d). Following a boost vaccination, both RBD-specific IgG titers and neutralizing GMTs in all adjuvant groups increase in magnitude (Figure 2c-d). MA103 induced a high SARS-CoV-2 neutralizing GMT of 3,446, which was comparable to that of MF59-like (3,000) and significantly higher than those of Al(OH)_3_ (456) and Al(OH)_3_ MPs (1,223) (Figure 2c-d). To characterize cellular immune responses induced by RBD-trimer formulated with Al(OH)_3_, Al(OH)_3_ MPs, MF59-like, or MA103, spleens of immunized mice were harvested at two weeks post the boost vaccination in another study and were then subjected to ICS assays. Upon re-stimulation with RBD peptide pools, SARS-CoV-2-RBD-specific CD4+ T cells elicited by the MA103 group exhibited significantly higher Th1 (IFN-γ, IL-2, and TNF-α) and Th2 (IL-4) cytokine ratios than those of placebo and Al(OH)_3_ MPs groups (Figure 2e and S3), indicating that when adjuvanted with MA103, RBD-trimer elicited a robust Th1/Th2 balanced cellular immune response. To directly verify the stability of RBD-trimer present in different adjuvant formulations, melting temperatures (Tm) of RBD-trimer were assayed using DSF. RBD-trimer in MA103 formulation exhibited a higher Tm value (51.4°C) than those in MF59-like (49.3°C) and PBS (48.9°C) formulations, representing a more stable state of the antigen (Figure S4a). Taken together, RBD-trimer adjuvanted with MA103 resulted in increased protein stability and optimal immunogenicity.

### Durability of humoral immune responses following SARS-CoV-2 RBD-trimer vaccination

**Figure 3:**
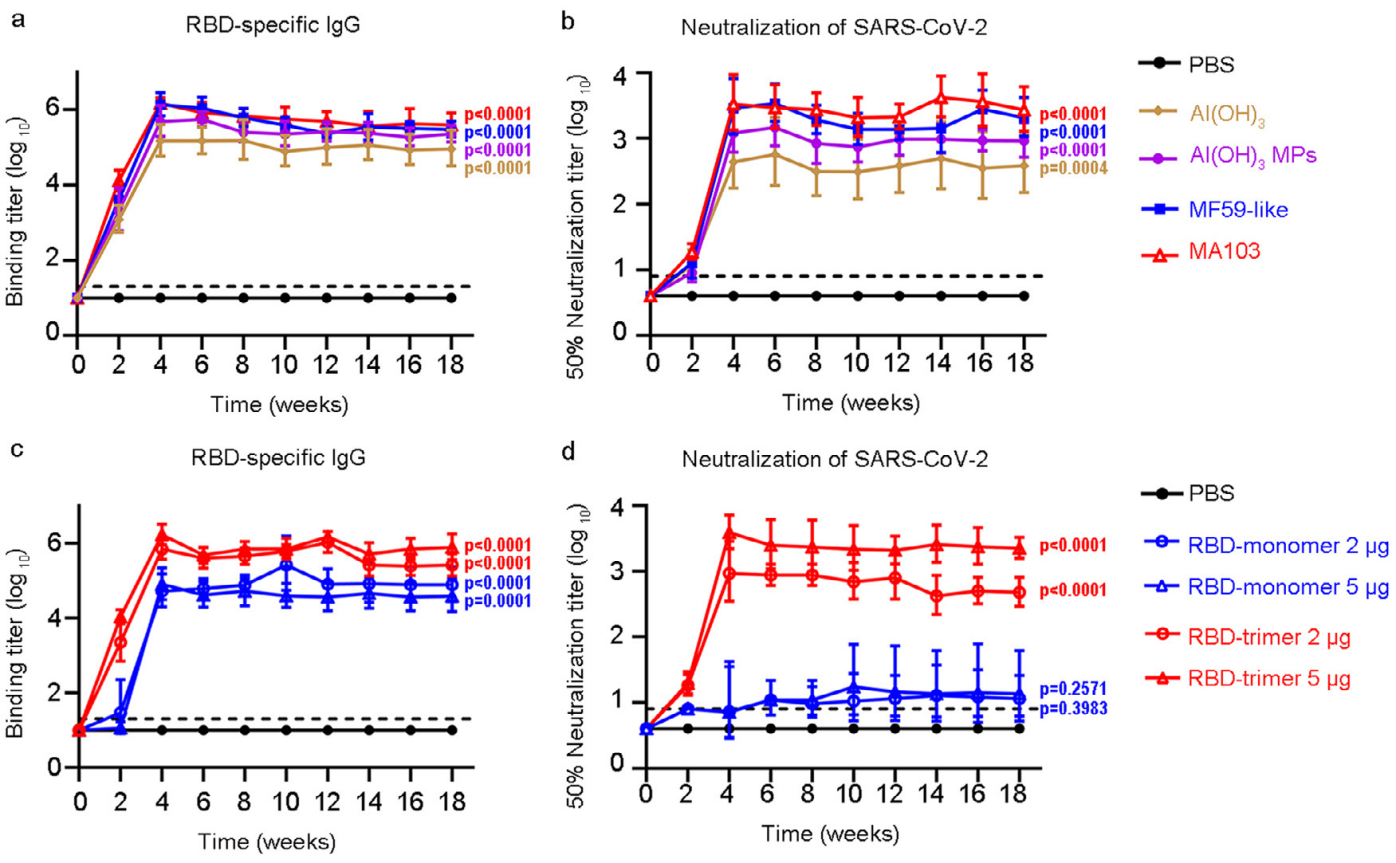
**Long-term monitoring of humoral immune response induced by RBD-trimer in mice. a-b,** Groups of 8-week-old female BALB/c mice (*n* = 5 per group) were employed to assess the persistence of RBD-trimer vaccine with various adjuvants. **c-d,** Groups of 8-week-old female BALB/c mice (*n* = 5 per group) were employed the consistent immunization strategy with RBD-based vaccine. SARS-CoV-2 neutralization and RBD-specific IgG titer assays were performed to assess the persistence of RBD-monomer and RBD-trimer vaccines in MA103 formulation. The values are representative of mean ± SEM. P values were analyzed with ordinary one-way ANOVA.

To investigate the long-term humoral immunogenicity of RBD-trimer, we performed a time-course study of IgG and neutralizing GMTs in sera of mice inoculated with two injections of placebo or 5 µg RBD-trimer in combination with one of those above adjuvants. In contrast to placebo, all RBD-trimer groups exhibited similar time-course patterns for both IgG and neutralizing titers, characterized by a moderate and sharp titer increase following the prime and boost vaccination, respectively, and then the persistence of peak titers until week 18 (Figure 3a-b). In addition, the MA103 group exhibited the highest IgG and neutralizing titers at all time points (Figure 3a-b). Another time-course study of humoral response in mice receiving RBD-monomer (2 or 5 µg) or RBD-trimer (2 or 5 µg), both adjuvanted with MA103, demonstrated that in contrast to the potent ability of RBD-trimer to induce long-lasting and high-titer IgG and neutralizing antibodies against SARS-CoV-2, RBD-monomer only elicited neutralizing GMTs around the detection limit of a titer of 1:8 and lower IgG titers over the entire study period (Figure 3c-d).

### Characterization and immunogenicity of H1N1 influenza HA1-trimer vaccine candidate

**Figure 4:**
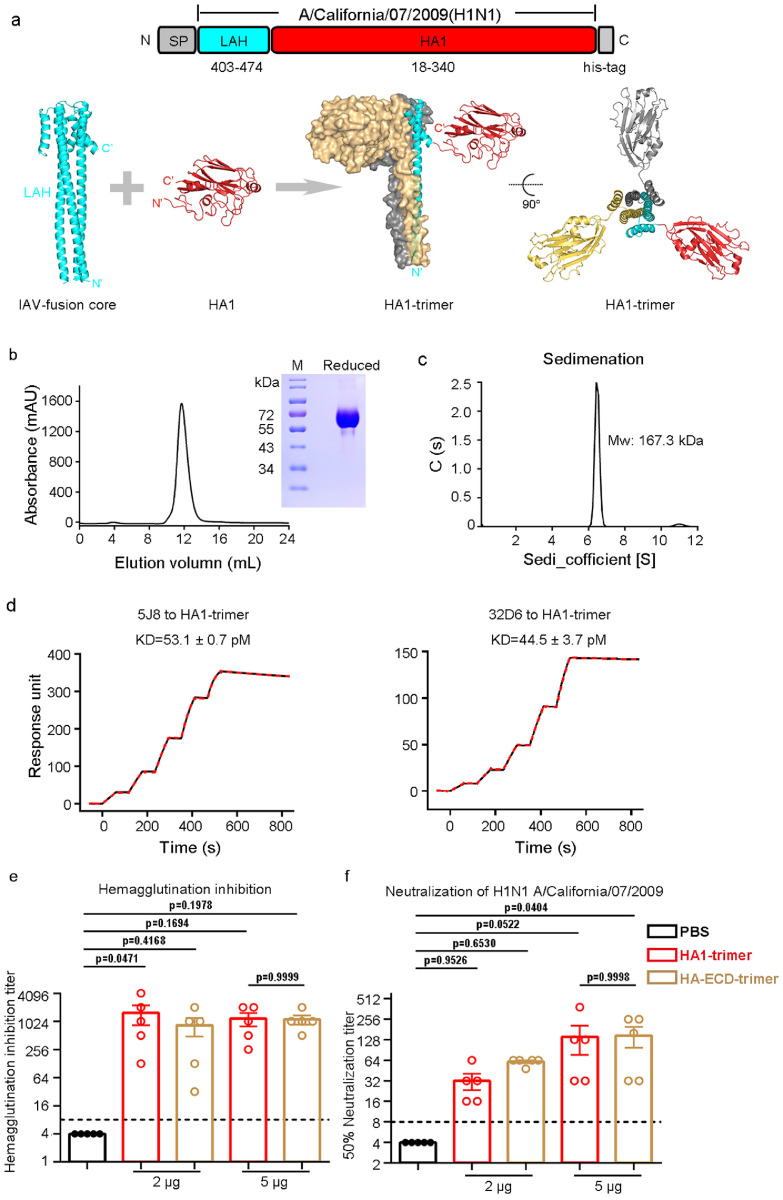
**Construction and characterizing of the trimeric HA1 vaccine candidate. a,** Schematic diagram of linking the IVA H1N1 A/California/07/2009-fusion core and HA1 to form a trimeric molecule. The upper panel shows the construction of the HA1-trimer. The lower panel shows the expected structures of the assembled trimeric molecule. **b,** Size-exclusion chromatogram and SDS-PAGE analyses of HA1-trimer. The cell supernatant was sequentially purified with Histrap excel and Superdex 200 Increase 10/300 GL column in PBS. The molecular in the single peak of gel filtration profile was shown in reducing SDS-PAGE. **c,** Ultracentrifugation sedimentation profiles of HA1-trimer. **d,** The binding kinetics profiles of HA1-specific MAbs to trimeric HA1 were assessed using a single-cycle model. MAb 5J8 or 32D6 protein was captured on the chip while serial dilutions of HA1-trimer protein then flowed over the chip surface. The kinetic parameters were labeled accordingly. Values are representative of mean ± SEM of three independent assessments. **e-f,** Groups of 8-week-old female BALB/c mice (*n* = 5 per group) were immunized with two-dose MA103-adjuvanted HA1-trimer or HA-ECD-trimer. PBS formulated with adjuvant was given as control. 14 days post the 2nd immunization, the serum of mice was collected to evaluate the humoral response to antigens. HAI and influenza virus neutralization assays show hemagglutination inhibition titers (e) and neutralization titers (f). The values are representative of mean ± SEM. P values were analyzed with ordinary one-way ANOVA.

Next, we expanded the strategy of non-tagged trimerization of RBD to vaccine design against IAV. We constructed HA1-trimer by fusing in tandem the conserved post-fusion LAH with HA1, both from H1N1 A/California/07/2009 influenza strain (Figure 4a), similar to the design of SARS-CoV-2 RBD-trimer (Figure 1a). Transient transfection of HEK293T cells enabled high lab-scalable production of soluble HA1-trimer (11.7 mg/L) (Figure S1b-c). SDS-PAGE analysis displayed a larger MW of monomeric subunit in HA1-trimer than its theoretical value (45 kDa), implying glycosylation modifications (Figure 4b). Analytical ultracentrifugation determined HA1-trimer MW as 167.3 KDa, suggesting a stable trimer formation (Figure 4c). Interestingly, SPR analysis demonstrated relatively higher binding affinities of HA1-trimer to two non-overlapping HA1-targeting monoclonal antibodies, 5J8 (KD = 53.1 ± 0.7 pM) and 32D6 (KD = 44.5 ± 3.7 pM) (Figure 4d), than those of HA extracellular domain (HA-ECD-trimer) to 5J8 (KD < 5 nM) and of HA1-monomer to 32D6-Fab (KD = 353 pM) in previous reports,^34^^,^^35^ suggesting that HA1 is correctly folded in HA1-trimer and can bind receptor binding site (RBS)-specific antibodies. To assess the immunogenicity, we immunized BALB/c mice (*n* = 5 per group) with HA1-trimer or HA-ECD-trimer (2 or 5 µg) in two-dose regimens. PBS was given as a negative control. A prime immunization induced undetectable HAI titers in all groups (data not shown). Following the boost immunization, HA1-trimer induced robust comparable HAI and neutralizing titers against H1N1 A/California/07/2009 influenza to HA-ECD-trimer at both doses (Figure 4e-f). In contrast, no HAI and neutralizing antibodies were detected in mice vaccinated with PBS (Figure 4e-f). HA1 subunit was also immunodominant as evidenced by 90% of HA1-trimer-specific IgG targeting it (Figure S2b). Additionally, the formulation improved protein thermal stability of HA1-trimer as shown in the DSF assay (Figure S4b). Thus, those results confirmed the stable trimer assembly of HA1 in HA1-trimer immunogen and its potent immunogenicity.

### Protection efficacy of a combination vaccine candidate against SARS-CoV-2 and IAV challenge

**Figure 5:**
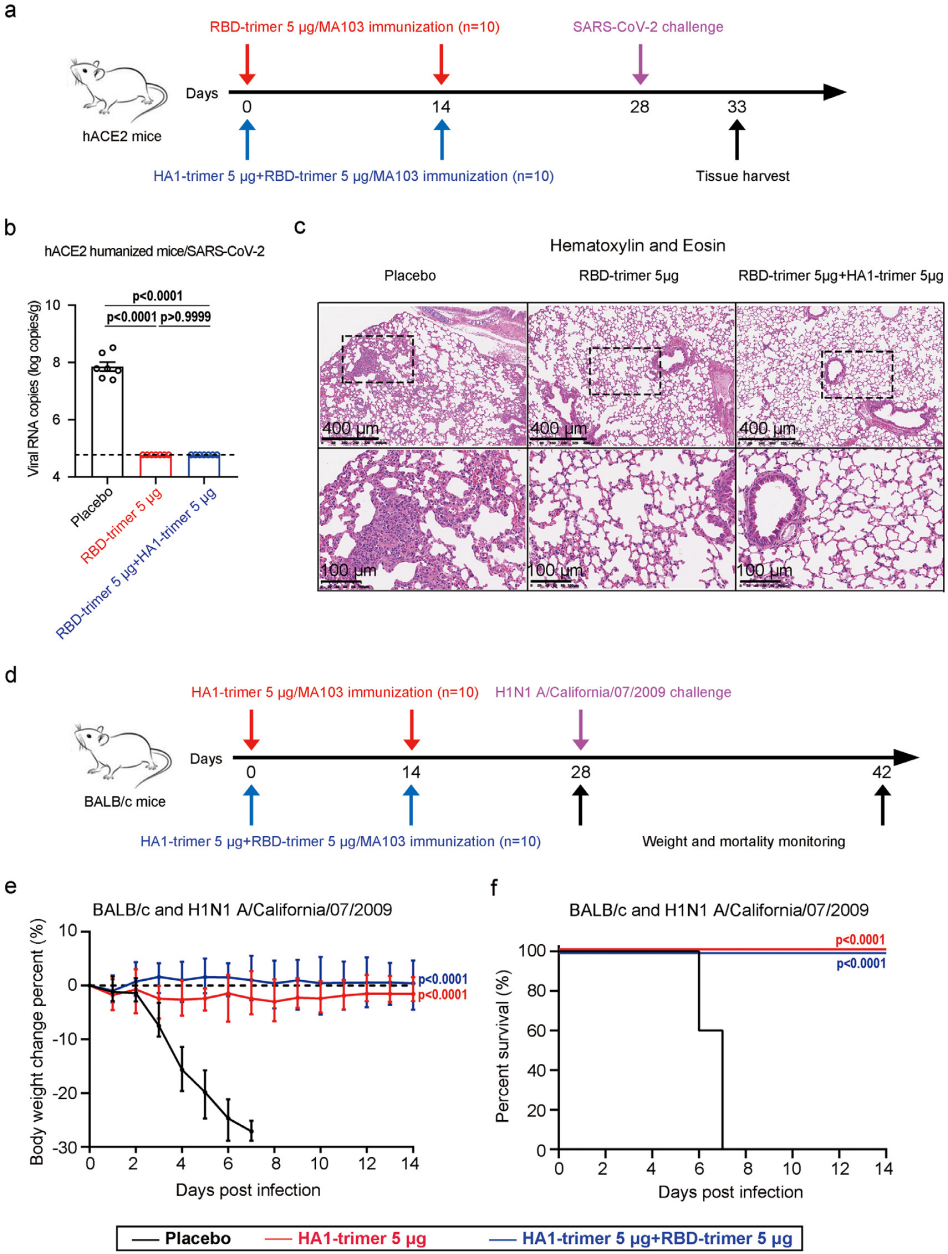
**Protective efficacy of a combination vaccine candidate against SARS-CoV-2 or IVA challenge in mice. a,** Experimental schematic of vaccines immunization and SARS-CoV-2 challenge. Groups of 14-week-old hACE2 mice (*n* = 10 per group) were immunized twice with vaccines and inoculated with 5 × 10^5^ TCID_50_ SARS-CoV-2 virus. **b,** The lung tissues were collected on the 5th day post-infection and RT-qPCR was used to measure viral RNA in lung tissues. The values are representative of mean ± SEM. P values were analyzed with ordinary one-way ANOVA. **c,** Hematoxylin and eosin staining sections exhibited histopathological changes in lung tissues. The scale bar in the upper and lower panel is 400 and 100 μm. **d,** Experimental schematic of vaccines immunization and pandemic influenza virus challenge. Groups of 8-week-old BALB/c mice (*n* = 10 per group) were immunized twice with vaccines and inoculated with 1 × 10^4.8^ TCID_50_ H1N1 A/California/07/2009 virus. **e-f,** Changes of body weight and survival curves of mice in 2 weeks later by challenging with influenza virus. Error bars denote SEM of the mean. For weight loss (e) and survival curves (f), asterisks indicate significance compared to the placebo group by ordinary one-way ANOVA and Mantel-Cox log-rank test.

A combination vaccine against SARS-CoV-2 and H1N1 influenza was prepared by mixing RBD-trimer with HA1-trimer at either dose of 5 µg. Next, we explored the *in vivo* protection efficacy of the combination vaccine against wild SARS-CoV-2 and H1N1 influenza challenges using hACE2 transgenic mice and BALB/c mice, respectively. Female hACE2 transgenic mice (*n* = 10 per group) were inoculated with two injections of PBS, RBD-trimer (5 µg), or the combination vaccine at a two-week interval (Figure 5a). At 28 days following the prime vaccination, all hACE2 transgenic mice were challenged with 5 × 10^5^ TCID_50_ of SARS-CoV-2 via the intranasal route (Figure 5a). Mice were euthanized at five days post-infection (dpi), and lung tissues were harvested for virus titer detection (*n* = 7 per group) and histopathological examination (*n* = 3 per group) (Figure 5a). A high level of viral RNAs were detected in the lungs of mice in the placebo group (∼10^8^ RNA copies equivalents per gram), indicating the viral replication status (Figure 5b). In contrast, nearly undetectable viral RNAs (∼10^4.7^ RNA copies equivalents per gram) were present in all mice vaccinated with the individual RBD-trimer vaccine or the combination vaccine (Figure 5b). Consistent with the virus load results, histopathological assays indicated that severe bronchopneumonia and interstitial pneumonia were observed in placebo mice, with edema and bronchial epithelial cell desquamation and infiltration of lymphocytes within alveolar spaces, whereas no such pathological changes were seen in mice lungs from both the individual RBD-trimer vaccine and the combination vaccine groups (Figure 5c). We also compared the serum RBD-binding IgG titers of vaccinated mice pre- and post-SARS-CoV-2 challenge. Consistent with high RBD-specific IgG titers (∼10^6^) before the challenge, all RBD-trimer vaccinated mice showed no significant increase in IgG titers, whereas in the placebo group there was a significant increase in IgG titers following SARS-CoV-2 challenge (Figure S5). These results demonstrated that two-dose administrations of the individual RBD-trimer vaccine or the combination vaccine efficiently prevented SARS-CoV-2 replication and protected mice from lung lesions. Similarly, female BALB/c mice (*n* = 10 per group) were immunized with PBS, HA1-trimer (5 µg), or the combination vaccine in a prime-boost regimen, and then were challenged with homologous H1N1 A/California/07/2009 influenza strain 28 days following prime vaccination (Figure 5d). All mice receiving the placebo control succumbed to virus challenge by 8 dpi, with severe weight loss starting from 2 dpi, whereas all mice receiving the individual HA1-trimer vaccine or the combination vaccine showed no mortality or morbidity (Figure 5e-f), indicating a high protection efficacy.

**Figure 6:**
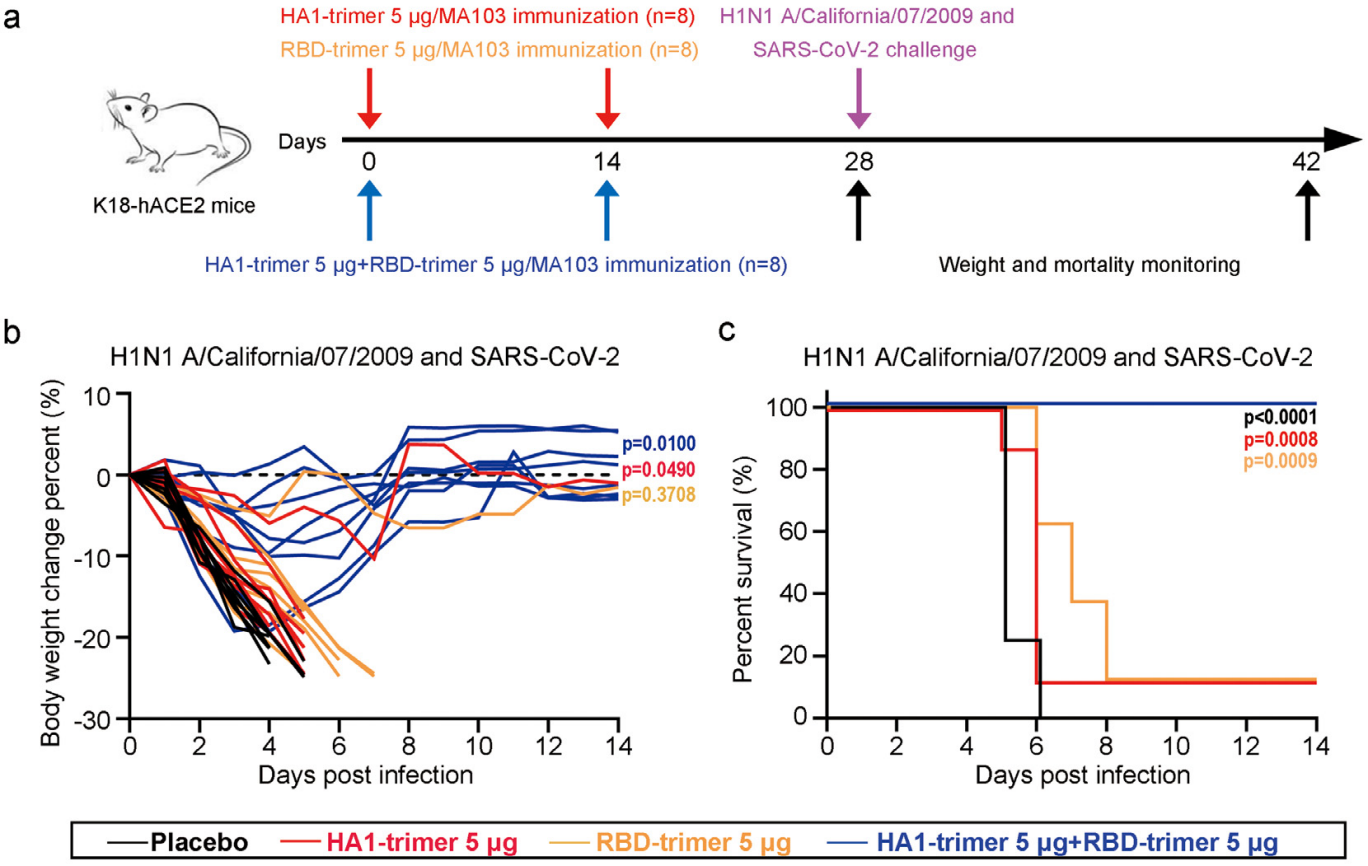
**Protection of a combination vaccine candidate against SARS-CoV-2 virus and IVA virus co-infections in the K18-hACE2 mouse model. a,** Experimental schematic of vaccines immunization and viruses challenge. Groups of 8-week-old K18-hACE2 mice (*n* = 8 per group) were immunized twice with single or combined vaccines and inoculated with 1 × 10^5^ TCID_50_ IAV H1N1 A/California/07/2009 and 1 × 10^2^ TCID_50_ SARS-CoV-2 virus. **b-c,** Changes of body weight and survival curves of mice in two weeks later by challenging with influenza and SARS-CoV-2 virus co-infections. For weight loss (b) and survival curves (c), asterisks indicate significance compared to the placebo group by ordinary one-way ANOVA and Mantel-Cox log-rank test.

As previously demonstrated,^6^ SARS-CoV-2 and IAV co-infection led to a greater risk of disease progression. The K18-hACE2 transgenic mice were used to study the potency of the single or combined vaccines against SARS-CoV-2 and IAV co-infection *in vivo* (Figure 6a). After two-dose administrations of a placebo, the individual RBD-trimer, or HA1-trimer vaccine, almost all of SARS-CoV-2 and IAV co-infected mice (8/8 or 7/8 in placebo- or single vaccine-treated groups) lost more than 25% body weight in 7 dpi (Figure 6b). In stark contrast, the same titers of two viruses triggered only a temporary weight loss in combined vaccines immunized mice and the mice had a comparable profile to the weight before infections at 14 dpi (Figure 6b). Importantly, 100% of animals in the combined vaccines group survived compared to 13% in the individual vaccine group and none in the placebo group (Figure 6c), indicating a strong prophylactic protection effect of combined vaccines against stringent lethal challenges of SARS-CoV-2 and IVA co-infection.

## Discussion

A high-probability co-epidemic of SARS-CoV-2 and influenza highlights an urgent need for the development of a combination vaccine against both SARS-CoV-2 and influenza virus. In the present study, we reported a generalized non-tagged trimerization design of RBD for class I virus fusion protein assisted with fusion core in post-fusion conformation. The resultant immunogens, SARS-CoV-2 RBD-trimer and IAV H1N1 HA1-trimer, were developed as vaccines. In line with previous reports,^21^ SARS-CoV-2 RBD-trimer induced significantly higher neutralizing titers compared to RBD-monomer, RBD-dimer, or S-ECD-trimer. Similarly, HA1-trimer also exhibited potent immunogenicity and induced high HAI and neutralizing titers against homogenous H1N1 A/California/07/2009 influenza. More importantly, a combination vaccine candidate composed of RBD-trimer and HA1-trimer conferred excellent protection against both SARS-CoV-2 and lethal homogenous H1N1 influenza infections in mouse models, supporting its further clinical investigation.

Multiple COVID-19 recombinant subunit vaccines based on RBD and S protein have been developed and entered into clinical trials.^43^^,^^45^ Compared with S protein-based vaccine, the RBD-based vaccine takes several advantages. Firstly, S protein in pre-fusion conformation is metastable and difficult to produce recombinantly.^32^ Although variants with beneficial proline substitutions exhibited higher expression than their parental construct, large-scale production of pure S remains relatively challenging.^46^ By contrast, RBD is relatively stable and amenable to mass production. Secondly, SARS-CoV-2 variants have been emerging and circulating worldwide during COVID-19 pandemic. It has been shown that nearly all neutralizing antibodies induced by S immunogen targeted RBD or N-terminal domain (NTD).^47^ In contrast to anti-RBD neutralizing antibodies that recognize multiple non-overlapping epitopes, anti-NTD neutralizing antibodies appear to target a single supersite and are prone to lose in neutralization as a result of frequent mutations within the supersite occurred in SARS-CoV-2 variants, especially those variants of concern (VOCs).^47^, ^48^, ^49^ Thus, RBD-based vaccines can exhibit higher tolerance to SARS-CoV-2 variants compared to S-based or inactivated virus vaccines.^50^

In contrast to the high variability of the IAV HA1 subunit, the HA2 subunit, especially the stem region, is usually conserved between strains or even subtypes.^11^ Thus, various stem-directed monoclonal antibodies (mAbs), isolated from humans, mice, or phage display, exhibited largely higher neutralizing breadth compared to anti-HA1 antibodies.^11^ For example, MEDI8852, a human potent anti-stem neutralizing mAb, is able to neutralize all IAVs with a median half-maximal inhibitory concentration (IC_50_) value of 0.51 μg/mL, and provide superior therapeutic efficacy to that of oseltamivir in both mice and ferret models.^51^ Those antibody discoveries also revitalized hopes of developing a universal influenza vaccine by enhancing or focusing immune response to the conserved pre-fusion HA2 regions.^52^ However, those stem-binding mAbs such as CR6261, MEDI8852, and MHAA4549A currently suffered setbacks in clinical trials.^19^^,^^53^, ^54^, ^55^ CR6261 has shown limited efficacy.^19^ Both MEDI8852 and MHAA4549A failed to improve clinical outcomes over oseltamivir, and neither combination with oseltamivir provided additional benefit beyond that of oseltamivir alone.^53^, ^54^, ^55^ Moreover, there is increasing evidence that HA2-based vaccines can only induce a low and transient antibody response.^52^ By contrast, vaccine-elicited HA1-specific B-cell response, the aim of our HA1-trimer design, has been widely demonstrated to be very effective at protecting against target influenza stains.^52^ Very recently, although rare, five mAbs that bound SARS-CoV-2 stem helix in pre-fusion S2 subunit was isolated from COVID-19 convalescent donors and showed broad but moderate neutralizing potency against beta-coronavirus *in vitro*.^56^ In principle, the use of fusion core in post-fusion conformation in our vaccine design was a suboptimal choice in inducing those above broadly neutralizing antibodies that usually target some pre-fusion epitopes or regions in S2 or HA2 subunit. However, the subdominant nature of influenza HA2,^52^ low frequencies (0-2.5%) of those stem helix IgGs in COVID-19 convalescent patients,^56^ and the high stability of fusion core in post-fusion conformation warrant its employment in vaccine design.

Our mice studies demonstrated that MA103 is superior to Al(OH)_3_ and Al(OH)_3_ MPs in inducing neutralizing antibody and cellular immune responses. However, we did not observe a significant difference between MA103 and MF59-like although there was a trend for better immune outcomes for MA103. The non-significant differences between MA103 and MF59-like could be due to the high dose of RBD-trimer (5 µg) in the study. NVX-CoV2373, an advanced clinical vaccine candidate that utilizes similar saponin-based adjuvant Matrix-M™, exhibited favorable safety profiles and comparable efficacy against SARS-CoV-2 to that seen with mRNA vaccines.^43^^,^^57^^,^^58^ Undoubtedly, those NVX-CoV2373 data supported our selection of MA103 as a vaccine adjuvant. Moreover, our data demonstrated that MA103-adjuvanted RBD-trimer at a dose of 5 µg elicited high neutralizing antibody levels (GMT of 3,959), which was similar to those of mRNA-1273 (3,481)^59^ and BNT162b2 (1,689)^60^ and higher than those of adenovirus-vectored AZD1222 (5-40)^61^ and DNA vaccine (74-170)^62^ in animal models. Immunization with HA1-trimer vaccine also elicited HAI antibody titers of approximately 1024, which was at least 2-fold higher than those from the licensed trivalent inactivated vaccine in mouse models.^63^ Those data predict high levels of vaccine protective efficacy in further clinical trials.

Commonly, and the results of live virus-challenging 6-8 weeks post-boosting for mice, a minimum interval to allow plasma blasts for contraction, represent actual vaccine efficacy against viruses *in vivo*. Whereas, the serum assays demonstrated that RBD-trimer induces long-lasting and high-titer neutralizing antibodies against SARS-CoV-2 for over 18 weeks in the mouse model (Figure 3). Due to these immunized characteristics, there was no significant difference in efficacy assessments between 2 and 6-8 weeks after boost immunization in mice.

While our study characterizes and validates the potency of a combined vaccine against SARS-CoV-2 and IAV co-infection, it has several limitations. Firstly, the mechanisms of RBD-trimer eliciting a more powerful humoral immune response are not fully disclosed. The data presented here show that trimeric RBD-based vaccines induced higher neutralizing titers than the S-ECD-trimer in mice that received the same dose of antigen. Due to the lower molecular weights, the same dose of the RBD-trimer immunogen including more molar amount is one possible reason for potency. Notwithstanding this possibility, using the mass unit to compare the immunogenicity of various vaccines is acceptable. Secondly, the challenging experiments in our study were based on the RBD-trimer vaccine due to the higher neutralizing titers *in vitro*. Real-world studies confirmed immunizations of RBD- or S-based vaccines are powerful countermeasures to combat the COVID-19 epidemic.^64^^,^^65^ Additional *in vivo* comparison of our trimeric RBD and existing S-based vaccines may provide evidence of which is superior protection. Thirdly, we did not perform antibody neutralization assays against SARS-CoV-2 virus variants and other subtypes of influenza viruses. The emergence of novel SARS-CoV-2 strains carrying key residual substitutions on the S and RBD can significantly decrease the efficiency of approved vaccines. Yearly, WHO updates vaccines to prevent seasonal influenza epidemics. Whether our vaccine is able to protect against SARS-CoV-2 VOCs and novel IAV subtypes will need to be elucidated by follow-up studies. Moreover, recent studies reported that intranasal infection of SARS-CoV-2 variants frequently spread to and within the central nervous system in K18 ACE2 transgenic mice.^66^ It would be interesting to know if SARS-CoV-2-challenged K18 ACE2 transgenic mice were protected from virus infection-induced impairment of neurological functions by using the RBD-trimer vaccine.

In conclusion, our data indicated that the combined RBD-trimer and HA1-trimer vaccine candidate is a potential intervention for COVID-19 and pandemic influenza co-infection and deserves further translational development.

## Contributors

R.S., Y.G.Y., and J.Y. initiated and coordinated the project. R.S. and J.Y. designed the experiments. R.S., J.Z., and L.X. conducted the experiments with the assistance of X.D., F.W. Y.W., Z.W., D.Y., and Q.H. R.S., Q.H., and J.Y. analyzed the data and wrote the manuscript.

## Data sharing statement

Data reported in this paper will be shared by the lead contact upon request. This paper does not report original code.

## Declaration of interests

R. Shi, Q. Huang, and J. Yan are listed as inventors on pending patent applications for RBD-trimer and HA1-trimer. The other authors declare that they have no competing interests.

## Appendix Supplementary materials

Image, application 1
