# Supplementary material for: A combination vaccine against SARS-CoV-2 and H1N1 influenza based on receptor binding domain trimerized by six-helix bundle fusion core
Source: eBioMedicine. 2022 Oct 4;85:104297. doi: 10.1016/j.ebiom.2022.104297 (PMC9530591; doi:10.1016/j.ebiom.2022.104297)
Supplement: Supplementary file 1 [file mmc1.docx]

**Supplementary Information**

**A combination vaccine against SARS-CoV-2 and H1N1 influenza based on receptor binding domain trimerized by six-helix bundle fusion core**

Rui Shi, Jiawei Zeng, Ling Xu, Fengze Wang, Xiaomin Duan, Yue Wang, Zheng Wu, Dandan Yu, Qingrui Huang, Yong-Gang Yao, and Jinghua Yan

**Supplementary information, Figures S1-5.**

**Figure S1. Analysis of protein production by DAS-ELISA.**

**Figure S2. Biased binding of trimeric vaccine-induced serum.**

**Figure S3. Flow cytometry assays of intracellular cytokines in CD4+ T cells from immunized mice.**

**Figure S4. The effect of MA103 and MF59-like adjuvants on trimer stability.**

**Figure S5. RBD-binding IgG titers of pre- and post-challenge serum in mice.**


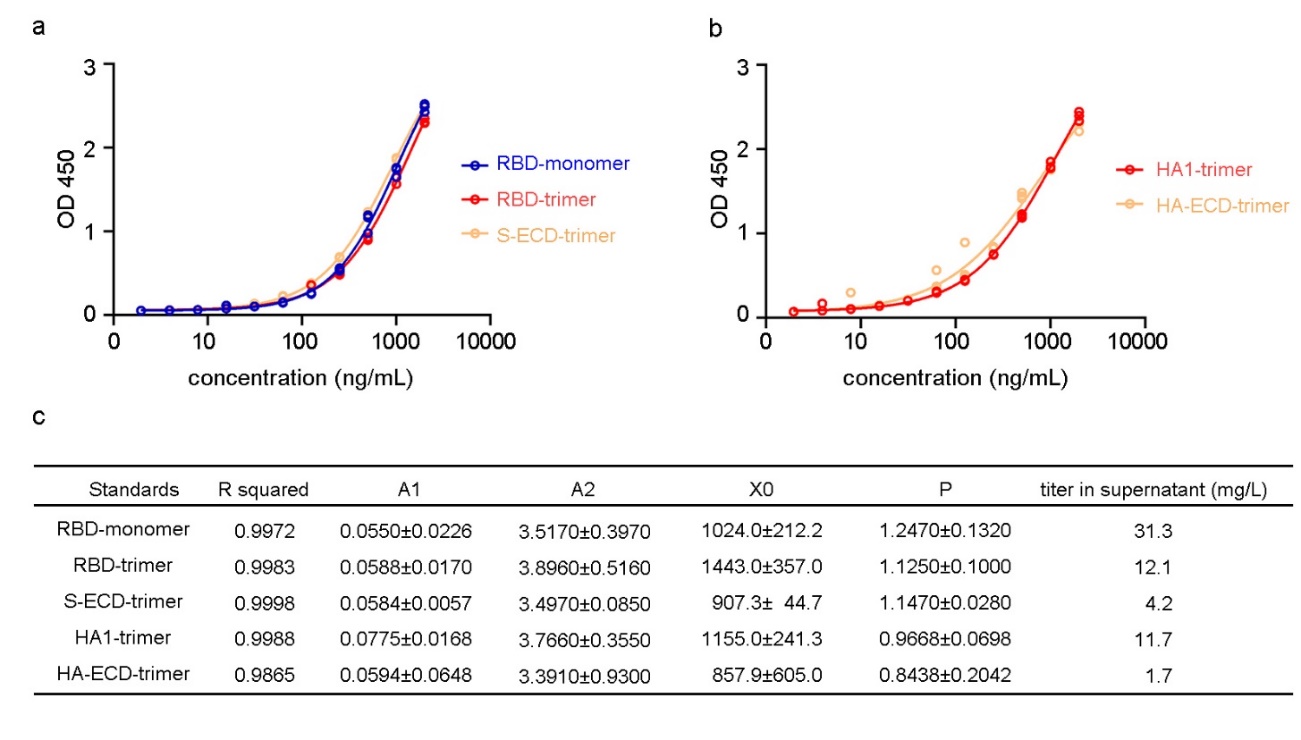
**Figure S1. Analysis of protein production by DAS-ELISA. a-b,** 4-PL curves of the antigen standards. The 4-PL curves were plotted by fitting concentrations of the RBD-monomer, RBD-trimer, S-ECD-trimer, HA1-trimer, and HA-ECD-trimer proteins on the X-axis and OD values on the Y-axis. **c,** The characteristics of two 4-PL curves are listed, A1 indicated the estimation of asymptotes under curves. A2 referred to the estimation of asymptotes on curves. X0 refers to the concentration for 50% of maximal effect and P represents the slope of the curve. [Equation: y=A2+(A1-A2)/(1+(X/X0)^P).


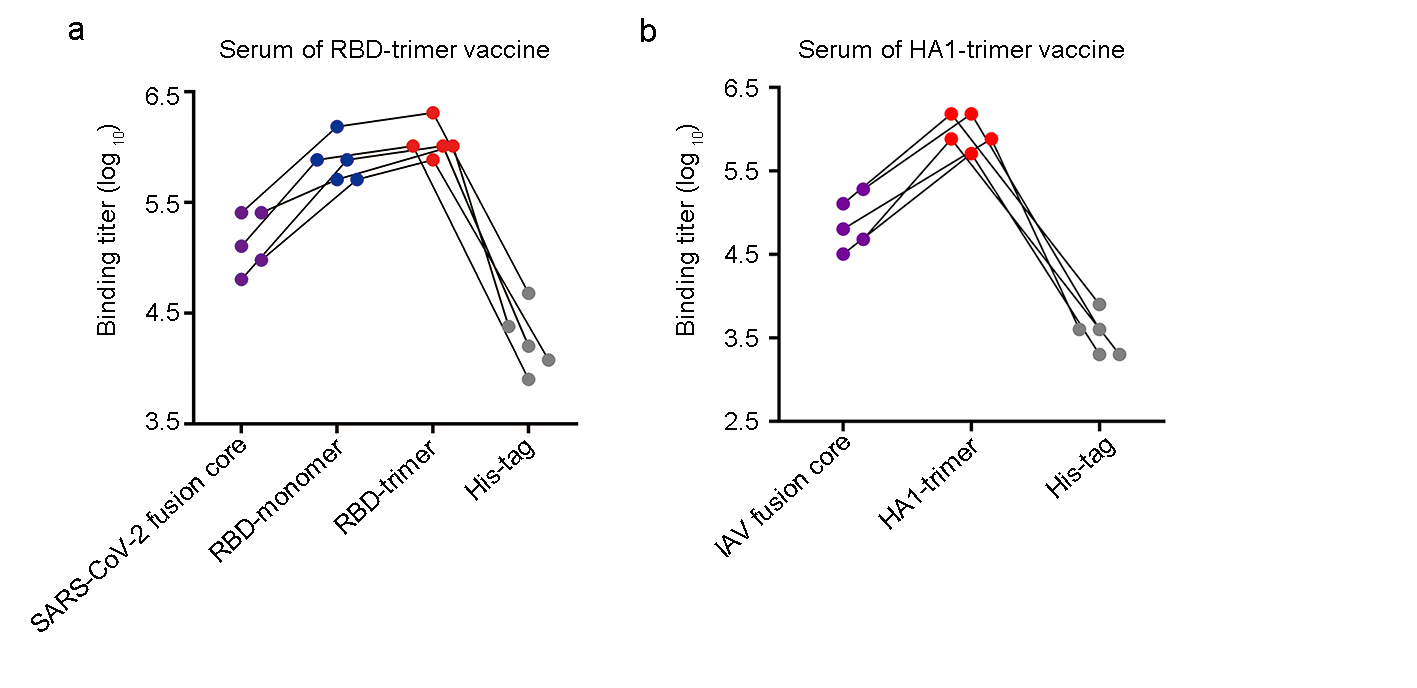
**Figure S2. Biased binding of trimeric vaccine-induced serum. a-b,** 14 days post 2nd immunization, the serum of mice (n=5) that received the trimeric vaccine was collected. Biased binding profiles of antibodies in the serum were tested using ELISA assays of coating fusion core, His-tag, monomeric antigen, and trimeric antigen proteins.


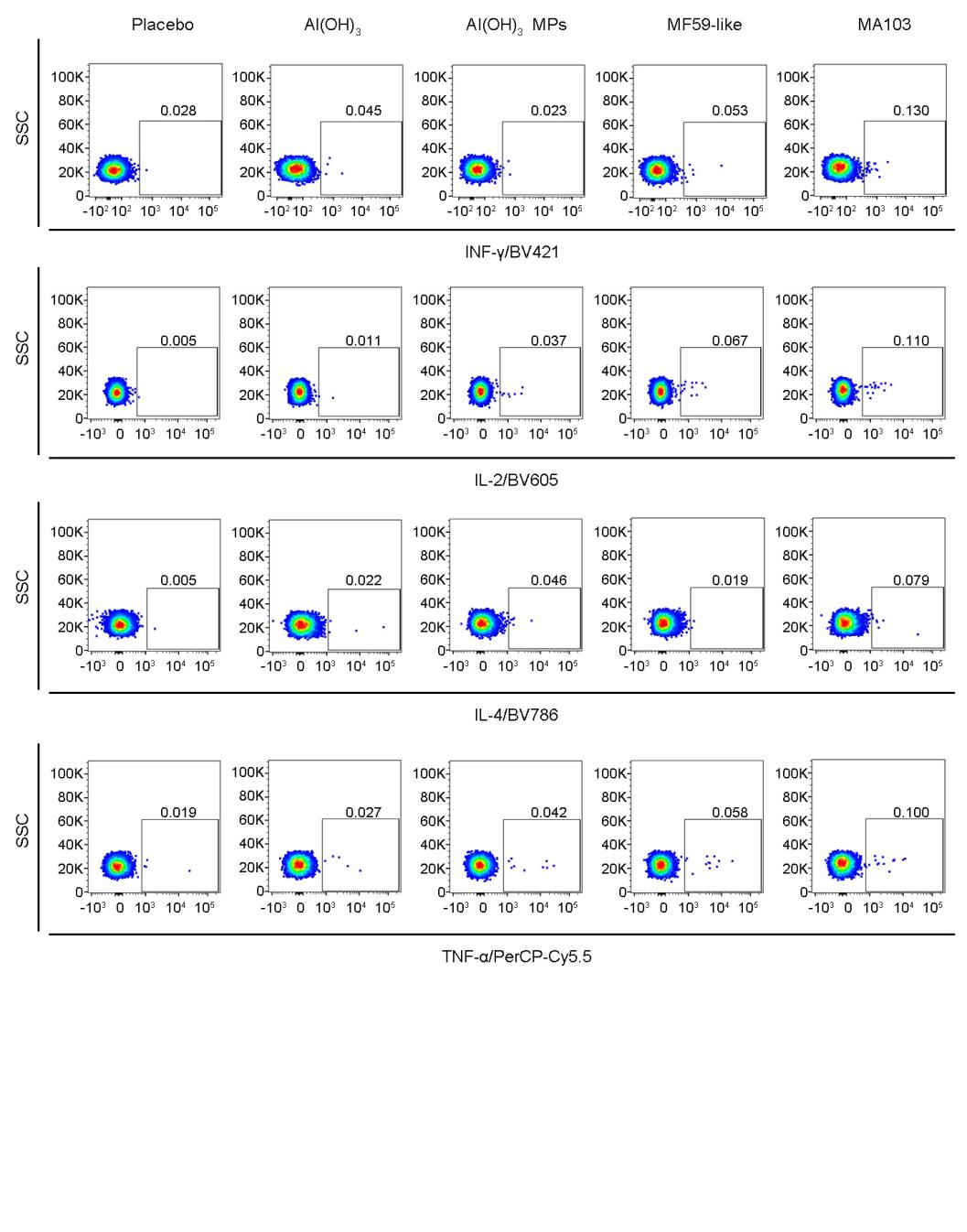
**Figure S3. Flow cytometry assays of intracellular cytokines in CD4+ T cells from immunized mice.** Splenocytes of adjuvanted RBD-trimer immunized mice were permeabilized after fixation. The cells were stained with anti-CD3, anti-CD4, and cytokines-specific fluorescent antibodies and assayed by flow cytometry. Experiments were performed twice and one representative data were displayed.


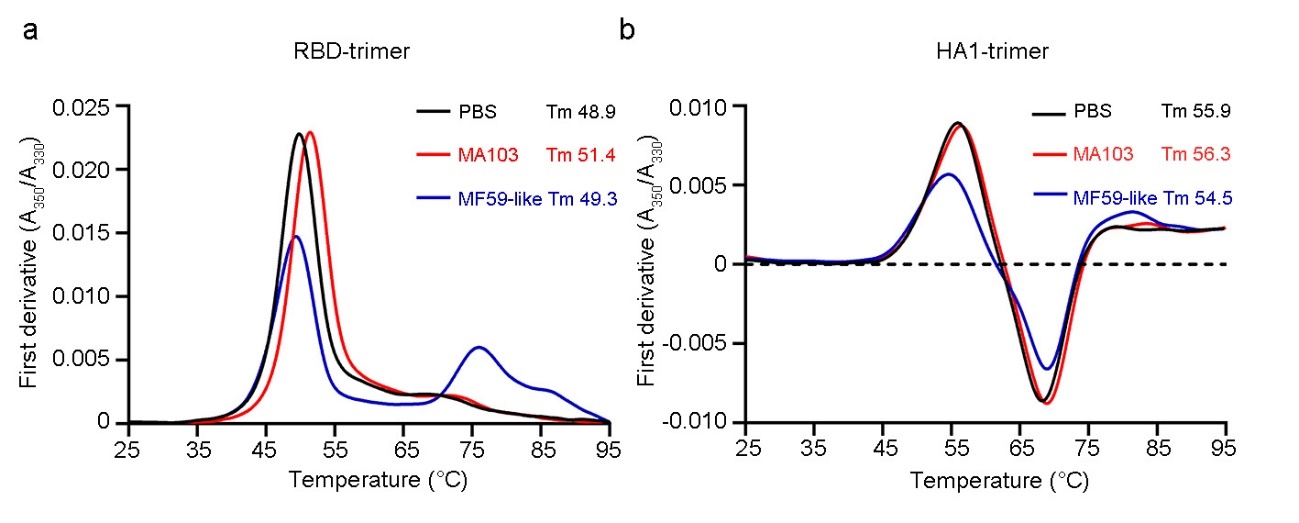
**Figure S4. The effect of MA103 and MF59-like adjuvants on trimer stability. a-b,** RBD-trimer or HA1-trimer protein was adjuvanted with MF59-like, MA103, or PBS at a working concentration of 1 mg/mL. The thermostability of trimers was derived from DSF analysis. The Tm values representing the mean of three independent assessments were labeled accordingly.


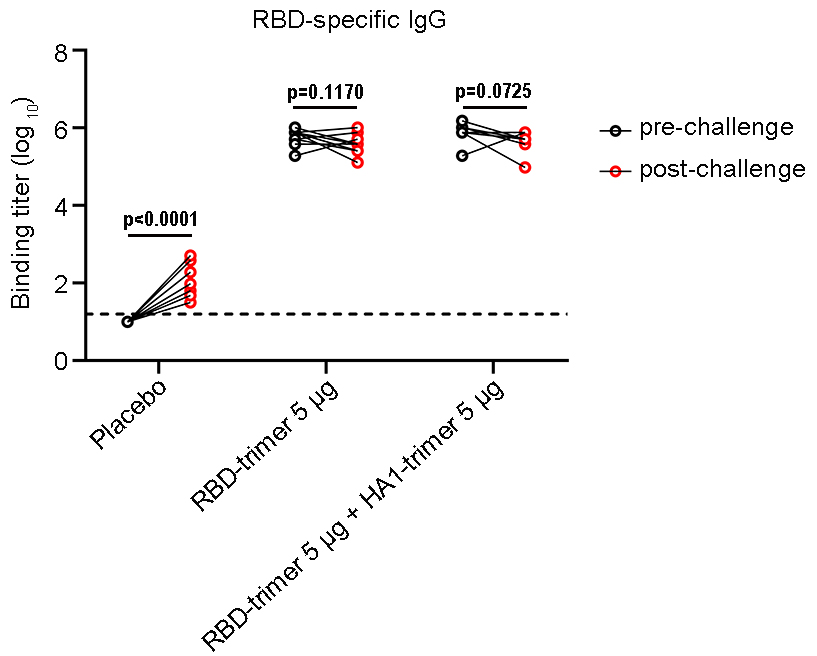
**Figure S5. RBD-binding IgG titers of pre- and post-challenge serum in mice.** The serum of mice (n=10) that received the trimeric vaccine or placebo was collected two days before and five days after SARS-CoV-2 challenge. Binding profiles of antibodies in the serum were tested using ELISA assays of coating monomeric SARS-CoV-2-RBD protein. P values were analyzed with t-test.
